# Belonging in their words: exploring early childhood perspectives using the draw, write, tell method

**DOI:** 10.1080/00049530.2025.2463949

**Published:** 2025-02-16

**Authors:** Kelly-Ann Allen, Emily Berger, Louise McLean, Erin Leif, William Warton, David Tuck, Marie Hammer, Marilyn Fleer

**Affiliations:** a School of Educational Psychology and Counselling, Faculty of Education, Monash University, Clayton, Victoria, Australia; b Well-being Science Centre, Faculty of Education, University of Melbourne, Parkville, Victoria, Australia; c Psychological Sciences, Faculty of Medicine, Nursing and Health Sciences, Monash University, Clayton, Victoria, Australia

**Keywords:** Belonging, qualitative, early years, preschool, safety

## Abstract

**Background:**

Belonging is a critical determinant of well-being in early childhood, yet empirical research on this concept for children aged 3 to 8 is limited. Aims: This study aimed to identify factors that promote belonging in children aged 3–8 to address the gap in existing research.

**Methods:**

The project involved the “Draw, Write, Tell” method to capture children’s perspectives on belonging through drawings and verbal responses. Parents and caregivers administered the survey to their children.

**Results:**

The study found that children’s understanding of belonging included themes such as happiness, safety, friendship, inclusion, and a caring environment. Unique aspects of belonging were noted between different age groups, highlighting developmental differences.

**Conclusion:**

This project provides a foundational basis for understanding belonging from the perspective of young children.

## KEY POINTS

**What is already known about this topic:**

1. Belonging is an important factor influencing well-being and development, yet research on young children aged 3 to 8 is limited.
2. Existing studies show the importance of inclusion, friendships, and positive interactions in fostering belonging in early educational contexts.
3. Early childhood frameworks, such as the EYLF in Australia, recognise belonging as essential but lack specific guidance for its practical implementation.

**What this topic adds:**

1. Children define belonging through themes like happiness, safety, friendship, and inclusion.
2. The study draws from children’s voices through the “Draw, Write, Tell” method, providing unique insights into belonging from their perspective.
3. Findings suggest that fostering belonging requires positive experiences and emotions.

Belonging, a fundamental human need, is defined in education as a student’s sense of being accepted, respected, and valued (Baumeister & Leary, 1995; Goodenow & Grady, 1993). Belonging has long been identified as a key factor impacting well-being, yet research specifically focusing on children aged 3 to 8 remains comparatively limited in scope. Despite policies prioritising belonging, there remains a need for more a concrete understanding of belonging in the early years to help early childhood educators and parents more effectively foster a sense of belonging in young children. The limited research to date has impeded the development of evidence-based practices, validated measures, and practical guidelines for this age group. Consequently, this study seeks to contribute to the existing literature by conceptualising belonging in children aged 3 to 8.

## Mental health and well-being

Childhood mental health serves as a predictor for overall well-being and quality of life throughout the lifespan, with implications for educational achievement, social bonds, career productivity, and even life expectancy (Productivity Commission, 2020). Moreover, the early childhood years are a critical developmental period, where adverse experiences exert a disproportionate impact on future mental health (Kuh, 2002; Langenberg et al., 2006). Positive experiences during this phase of life are particularly beneficial for later psychological adjustment (Lee & Schafer, 2021). A strong sense of belonging has been identified as essential for children’s development and long-term well-being (Scarf et al., 2016). Therefore, understanding how young children experience and conceptualise belonging is crucial. Exploring how belonging is constructed and defined in early childhood educational settings by young children themselves could offer actionable strategies and policies (Emilson & Eek-Karlsson, 2022; Johansson & Rosell, 2021). Such research has the potential to significantly affect not only childhood mental health but also broader dimensions of well-being throughout life.

## Belonging in early childhood

Belonging is an integral part of the Early Years Learning Framework for Australia (EYLF V2.0) and forms the core vision of the framework, emphasising the foundational role it plays in shaping a child’s early educational journey (Australian Government Department of Education [AGDE], 2022). The five key learning outcomes of the EYLF V2.0 encourage the promotion of children’s sense of identity, well-being, learning, communication skills, and connection with, and contribution to, their world. Belonging is particularly important for the identity and connection dimensions of the EYLF V2.0, which encourage the development of identity through safe and supportive connections with others in their environment. These connections help foster a sense of reciprocal rights and responsibilities, encouraging children to understand and engage with their community in meaningful ways. In this context, the concept of school belonging becomes crucial, especially during the transition from Early Childhood Education and Care (ECEC) to the early years of schooling. School belonging is defined as “the extent to which students feel personally accepted, respected, included, and supported by others in the school social environment” (Goodenow & Grady, 1993, p. 80). However, the role of belonging during the transition years from ECEC to early years of schooling (3–8 years) remains a largely underexplored avenue of research.

Previous studies have demonstrated the positive relationship between school belonging and various outcomes, including academic performance, psychological well-being, and physical health (Carter et al., 2007; Frydenberg et al., 2009; Ryzin et al., 2009). This underscores the significance of establishing a continuum of belonging from ECEC settings into the early years of formal schooling, as emphasised by the EYLF’s vision of Belonging, Being, and Becoming. Although the existing literature provides valuable insights, most research has focused on older age groups, with less emphasis on how younger children experience belonging in early education contexts. This gap is especially problematic given the prominence of belonging in the EYLF V2.0 and the National Quality Standards outlined by the Australian Children’s Education and Care Quality Authority (ACECQA). Both documents do not provide specific guidance on how belonging should be conceptualised or operationalised in early childhood education and care settings (Sumsion et al., 2018).

Although the EYLF V2.0 contains updates for suggested practices aimed at achieving better mental health outcomes in comparison to the original EYLF, it remains challenging to separate these practices from their stated outcomes. This absence of concrete guidance may result in challenges for educators aiming to promote belonging. While early childhood educators and teachers acknowledge the importance of fostering a sense of belonging in their pedagogical practices, they often report lacking the practical skills to implement this complex construct (Emilson & Eek-Karlsson, 2022). Given the complex, dynamic nature of belonging – which is shaped by individual experiences, social interactions, and environmental factors – early childhood educators need well-developed skills and strategies to engage with this concept effectively. A growing body of research has begun to explore the concept of belonging in early childhood settings (Einarsdottir et al., 2022; Johansson, 2017; Koivula & Hännikäinen, 2017). This study aims to build on these foundations by focusing on how young children directly express their feelings of belonging and identifying key factors that facilitate belonging in ECEC and early school contexts.

## Belonging research

Recent research has made strides in identifying factors that contribute to young children’s sense of belonging. Observational studies have highlighted the importance of cooperative communication between educators and children (Ree & Emilson, 2020), inclusion in collective activities (Johansson, 2017), and joint play, stable friendships, and emotional bonding (Koivula & Hännikäinen, 2017) in fostering a sense of belonging. Group interviews with early childhood educators revealed that feelings of togetherness, safety, integrity, and a caring approach enhance children’s belief in community. Additionally, an appreciation for diversity and learning from each other were deemed crucial for promoting belonging (Eek-Karlsson & Emilson, 2023; Gouldsboro, 2018). Qualitative studies across various countries found that friendships, caring adults, and community membership are vital for belonging in early education (Einarsdottir et al., 2022). However, approaches to belonging can vary significantly across different communities and contexts, highlighting the need for a clearer understanding of this concept (Johansson & Rosell, 2021).

## The current study

While research on belonging in early childhood is growing, more targeted research is needed to explore the experiences of children in ECEC settings and during the early years of school (3–8 years). This critical period is when many children are transitioning into various educational settings. This study aims to redress the gap in research by exploring how children aged 3 to 8 define and experience belonging. The study investigates children’s perspectives on belonging in ECEC and early school settings, identifying themes and factors that promote belonging from the children’s viewpoints. Subsequently, the current study is guided by the following research questions:

1. How do young children experience belonging in an ECEC and early years of school context?
2. What helps to facilitate young children’s belonging in an ECEC and early years of context?

## Method

### Sample

The sample consisted of 38 child participants spread across six age categories (see Table 1). To capture children’s perceptions, the study employed targeted sampling by recruiting parents and primary caregivers who were asked to administer the survey to their children aged 3–8 years. This approach aimed to enhance participant comfort and elicit richer responses, as parents and caregivers are well-positioned to understand their children’s expressions of belonging. Special attention was given to recruiting underrepresented groups. Table 1 shows the demographic characteristics of study participants.

**Table 1.** Participant demographics.

Variable	*n*	%
Age		
3	3	5.3
4	7	18.4
5	9	23.7
6	5	13.2
7	10	26.3
8	4	10.5
Prefer not to say	1	2.6
Gender^1^		
Female	24	63.2
Male	13	34.2
Non-Binary	-	-
Other	1	2.6
Prefer not to say	-	-
State of residence		
Victoria	26	68.9
Queensland	6	15.8
South Australia	3	7.9
New South Wales	1	2.6
ACT	1	2.6
Prefer not to say	1	2.6
Municipality		
Metropolitan	28	73.7
Regional	8	21.1
Remote/rural	2	5.3
Cultural background		
Anglo-Celtic	20	52.6
Multiracial/multiethnic	7	18.4
Asian	3	7.9
European	2	5.3
Middle Eastern	1	2.6
Pacific Islander	1	2.6
Australian	1	2.6
Aboriginal and Torres Strait Islander	0	0.0
Prefer not to say	3	7.9
Primary language		
English	30	78.9
Spanish	3	7.9
Punjabi	2	5.3
Arabic	1	2.6
Cantonese	1	2.6
Greek	1	2.6
ECEC and early years of school setting		
Primary School	20	52.6
Kindergarten/preschool	13	34.2
Centre based care	4	10.5
Prefer not to say	1	2.6
Neurodiversity		
ADHD	3	7.9
Autism	2	5.3
Autism and ADHD	1	2.6
Autism, dyslexia and dyspraxia	1	2.6
Symptoms of ADHD	2	5.3
None	29	76.3

*Note*: The demographic question regarding gender included response options that encompassed both biological sex (male, female) and gender identity (non-binary, other, prefer not to say). We acknowledge that this phrasing does not fully differentiate between sex and gender, and future research would benefit from separate measures for these constructs. Additionally, parents provided responses on behalf of their children, which may not accurately reflect children’s self-identified gender. The ‘Other’ option for gender identity included the text response “a boy from my perspective but identity is subjective so don’t feel confident to impose”. The neurodivergent status of participants, including ADHD, autism, and other conditions, was based on parent and caregiver self-report. The research team did not verify these diagnoses through official medical documentation. It is important to note that some caregivers may not be aware of their child’s neurodivergence, which could lead to either over- or under-reporting of neurodivergent participants in the sample.

### Methods

The current study was approved by Monash University Human Research Ethics Committee (ID: 39562). Data were collected through an online survey hosted on the Qualtrics platform. On the landing page of the survey, participants were provided with an explanation of the study’s focus on belonging in ECEC and early years of school settings. The following page presented the study’s explanatory statement, detailing the aims, the voluntary nature of participation, and the option for participants to withdraw from the study at any time. In addition, contact information for mental health services was provided in case participants experienced distress from the content of the study.

The current study adapted the Draw, Write, Tell methodology (Angell et al., 2015; Pope et al., 2019; Waters et al., 2022) to explore children’s perceptions of belonging. This approach provides a multidimensional lens for understanding how children express their thoughts and feelings through a combination of drawings, verbal narratives, and written responses.

**Figure 1.**
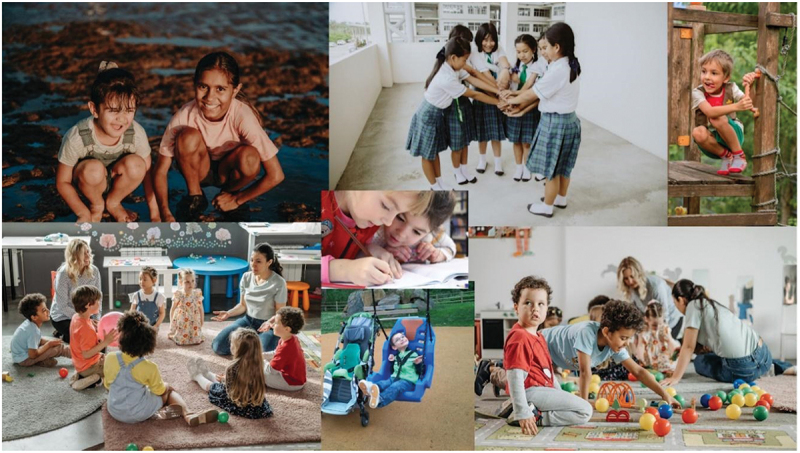
Pictures shown to participants depicting belonging.

Parents and primary caregivers were provided with a written description of the study procedure and a social script (see Supplemental) that introduced the concept of belonging. This script was designed to help children understand the idea of belonging through a relatable narrative. Alongside the script, parents presented their children with a series of culturally diverse pictures (see Figure 1) illustrating situations where children might feel a sense of belonging in their ECEC or early school environments. These visual aids were intended to support children’s comprehension of the subsequent questions. The pictures were selected based on our understanding of how young children (aged 3–8) may conceptualise belonging. These images were drawn from a previous project (Hudson & Allen, n.d.) which investigated children’s sense of school belonging during their first year of school. While the project is currently under review, the selection was guided by insights from this work, with the aim of ensuring that the images represented a broad and inclusive spectrum of contexts likely to resonate with children in this age group.

Following this, parents read four priming questions to their children, designed to engage them with the concept of belonging in a way that aligned with their cognitive and linguistic development. These questions included:

- “Have you felt like you belong before?”
- “Where are some of the places you feel like you belong?”
- “How does belonging make you feel?”
- “Can you explain what you think belonging means in your own words?”

The last primer question – “Can you explain what you think belonging means in your own words?” - also served to check the child’s understanding, designed to assess whether the child had grasped the concept of belonging after hearing the script and viewing the pictures, and their verbal responses were recorded by parents in the text boxes provided in the online survey.

In the next stage, participants were asked questions related to their sense of belonging in ECEC or school contexts. The central research questions were:

- “What do you think belonging means at ECEC or school?” (Parents were instructed to use the term most relevant to their child’s setting, such as daycare or kinder.)
- “What helps you feel like you belong at ECEC or school?”

Parents were also instructed to encourage their child to respond using their own words, rather than simply repeating phrases from the story. Children were given the optional opportunity to draw pictures representing their understanding of belonging (the Draw stage of the methodology). In addition to the drawings, parents recorded the child’s verbal explanation of the *image* (the Write/Tell stage), transcribing these explanations into the survey’s text boxes. Responses, including both written descriptions of verbal explanations and optional drawings, were uploaded via the Qualtrics platform for analysis by parents. This method allowed for both visual and verbal data collection, offering a multidimensional understanding of how children perceive belonging.

We acknowledge that using parents and primary caregivers as the primary data collection agents may introduce potential confounds related to parent characteristics and their willingness to engage in the study. However, given the age group of the participants (3–8 years), this approach was chosen to ensure children felt comfortable expressing their thoughts and feelings in a familiar and trusting environment. The role of parents and primary caregivers in facilitating the study allowed for a more natural and less intimidating context for children to articulate their experiences of belonging. Given the intimate nature of the topic, caregivers were more likely to foster an environment where children could freely express their understanding of belonging without fear of judgement or anxiety associated with unfamiliar adults. Children aged 3–8 years are still developing their communication skills, and caregivers, who are more familiar with their children’s individual ways of expressing themselves, are better positioned to prompt richer, more nuanced responses. This was particularly important for the Draw, Write, Tell methodology, which combines verbal and visual expression.

The social script used in this study to introduce the concept of belonging was designed to ensure that all children had a shared understanding of the topic. However, we acknowledge that this could potentially influence children’s responses. To mitigate this, we included a key question – “Can you explain what you think belonging means in your own words?” - to assess the child’s independent interpretation. Additionally, by combining verbal responses with visual data from drawings, we could examine how children conceptualised belonging beyond the primed script, providing a richer and more nuanced understanding. The design aimed to balance clarity in presenting the concept with minimising the influence of priming on children’s responses. Parents and primary caregivers were also provided with clear guidelines to help reduce bias, including instructions to avoid leading their children towards specific answers and to encourage open-ended responses.

### Planned analyses

Demographic data were exported to SPSS (IBM Corp, 2020) for quantitative analysis using frequency statistics. Verbal responses were analysed using reflexive thematic analysis (Braun & Clarke, 2021a, 2021b, 2024), following six phases: familiarisation with data, initial coding, searching for themes, reviewing and refining themes, defining themes, and writing up. The research team selected Braun and Clarke (2021a, 2021b, 2024) reflexive thematic analysis approach because it aligns with critical qualitative research principles, viewing knowledge as contextual, subjective, and inherently partial, with reflexivity being a key aspect of the process (Koro-Ljungberg & Cannella, 2017).

The use of reflexive thematic analysis complemented the Draw, Write, Tell methodology, as children’s drawings were interpreted alongside their verbal explanations to understand their conceptualisation of belonging. Each drawing was examined for symbolic representation, emotional expression, and the presence of social contexts (e.g., inclusion of peers, educators, family members). The research team integrated the visual and verbal data by comparing themes that emerged from both datasets. Where there were overlaps between the visual and verbal themes, these were consolidated into a combined theme. Where divergences occurred (e.g., a child’s drawing depicted one concept, but their explanation emphasised another), both aspects were considered, and reflection was used to explore these differences, ensuring a deeper, more nuanced interpretation.

In the spirit of reflexivity, the process of theme development was not seen as a neutral extraction of meaning from the data, but rather as an interactive process in which the researchers’ perspectives and interpretations shaped the themes (see Braun & Clarke, 2021a, 2021b, 2024). The themes were developed iteratively through multiple rounds of coding, revision, and discussion. During these discussions, the research team revisited the data to ensure that the themes reflected the complexity and nuances of the children’s responses. Given the fluidity and subjectivity inherent in this approach, it was essential to adopt a transformative and reflective stance throughout the analysis. The research team did not use inter-coder reliability measures, as this is not in line with the principles of reflexive thematic analysis, which emphasises the subjective and interpretive nature of the data (see Braun & Clarke, 2021a, 2021b, 2024). Instead, the analysis was conducted collaboratively, with researchers engaging in ongoing dialogue to ensure that the themes developed were coherent, comprehensive, and well-grounded in the data. Discrepancies in interpretation were resolved through reflective discussion and negotiation, recognising the inherently subjective nature of thematic analysis. Saturation for the qualitative analyses was determined once no new themes were being identified in additional participant data (Saunders et al., 2018). This approach ensured that the analysis was both rigorous and sensitive to the perspectives of the children involved.

#### Positionality

Our research team brings a diverse range of expertise and lived experiences that inform the approach to this study. The team includes scholars with backgrounds in psychology, early childhood education, resilience research, and behaviour analysis. Personal experiences related to cultural identity, family structures, socio-economic backgrounds, and professional work in diverse educational and community settings shape our perspectives on belonging. This diversity enriches the research process by ensuring a multi-faceted understanding of how belonging is conceptualised and facilitated among young children, as well as a reflexive approach to data interpretation. Our team’s collective experiences with vulnerable populations, trauma, and inclusive practices further enhance our sensitivity to the complexities of belonging in early childhood contexts.

## Results

Results for the priming questions are found in the supplemental material.

### Children’s understanding of belonging

To understand how young children experience belonging in an ECEC and early years of school context, we analysed children’s verbal responses and drawings to identify themes. We explored the conceptualisation of belonging from the child’s perspective, including themes related to emotional states (e.g., happiness, safety), social relationships (e.g., friendship, inclusion), and the sense of identity (e.g., feeling at home or being part of a group). These responses reflect children’s innate understanding of what belonging feels like and how they interpret the world around them in relation to belonging. While these responses reflect children’s understandings, it’s important to note that the conceptualisation of belonging as defined by children is often influenced by their environment and social contexts, which makes the section, *Factors Promoting Belonging*, a complementary lens to understand the external forces and interactions that shape this understanding.

#### Verbal responses

Analysis of participant responses uncovered eight overall themes related to participants’ understanding of belonging in education and care contexts. These Included: Positive Environment, Friendship, Fun/doing activities, Inclusion, Responsibility, Safety, Caring Environment, Feeling Comfortable as Home. Where participant responses involved more than one theme, their response was placed into all relevant themes. The verbal response themes and participant quotes are included in Table 2.

**Table 2.** Verbal response themes.

Positive environment (n = 15, 39.5%)	Friendship (n = 9, 23.7%)
‘Feeling good in your class and spaces’ (girl, age 8) ‘Feeling happy’ (girl, age 4) ‘Included and happy’ (girl, age 7) ‘It means that everyone is working and helping each other. Kids are nice to each other. Teachers are kind’ (boy, age 8) ‘Respect of your teacher elders and friends’ (boy, age 6) ‘Belonging means like where you are you happy or somewhere like that’ (boy, age 5) ‘To feel safe, calm and happy and you always know friends are there for you’ (girl, age 7) ‘You have to be kind and nice and listen to your teachers. That shows you are belonging at school. Make new friends’ (boy, age 6) ‘I love all the teachers, they make me feel safe and happy and funny’ (girl, age 5) ‘Everyone being nice and playing together’ (boy, age 5) ‘It means that we’re all part of the school, and we all follow the rules, be safe, respectful and being a learner’ (girl, age 8) ‘My teacher takes photos of our class together and it feels lovely to see them’ (girl, age 6) ‘That you’re happy in this place and you feel happy and excited when you go there’ (girl, age 7) ‘When everyone is nice to each other’ (boy, age 6) ‘When you feel happy at school. It makes you feel like you are home’ (girl, age 8)	‘Belonging means having friends’ (girl, age 5) ‘Playing with my friends’ (girl, age 5) ‘Respect of your teacher elders and friends’ (boy, age 6) ‘To feel safe, calm and happy and you always know friends are there for you’ (girl, age 7) ‘You have to be kind and nice and listen to your teachers. That shows you are belonging at school. Make new friends’ (boy, age 6) ‘Because I play with my friends and it’s fun and I like them’ (girl, age 5) ‘It means being with other people and always having fun’ (girl, age 7) ‘In it together with my friends’ (girl, age 8) ‘The stuff we do at school, my teacher and my friends’ (boy, age 6)
**Fun/Doing activities (*n* = 8, 21.1%)**	**Inclusion (*n* = 6, 15.8%)**
‘Doing activities’ (girl, age 3) ‘Have fun’ (girl, age 3) ‘Because I play with my friends and it’s fun and I like them’ (girl, age 5) ‘Everyone being nice and playing together’ (boy, age 5) ‘It means being with other people and always having fun’ (girl, age 7) ‘People listen to me and play with me and when I’m included’ (girl, age 7) ‘The stuff we do at school, my teacher and my friends’ (boy, age 6) ‘They make me feel happy by playing with me. They have toys that I like’ (girl, age 5)	‘Getting included’ (boy, age 7) ‘Included and happy’ (girl, age 7) ‘Where you are not behind, where you are loved’ (girl, age 4) ‘People listen to me and play with me and when I’m included’ (girl, age 7) ‘They make me feel happy by playing with me. They have toys that I like’ ‘When people choose me’ (girl, age 7)
**Responsibility (*n* = 5, 13.2%)**	**Safety (*n* = 4, 10.5%)**
‘You have to be kind and nice and listen to your teachers. That shows you are belonging at school. Make new friends’ (boy, age 6) ‘Everyone has to look after each other’ (boy, age 4) ‘It means that everyone is working and helping each other. Kids are nice to each other. Teachers are kind’ (boy, age 8) ‘Being kind and letting other people play your games. Not being too rough and just having other people with you’ (girl, age 7) ‘It means that we’re all part of the school, and we all follow the rules, be safe, respectful and being a learner’ (girl, age 8)	‘I be safe and the teachers keep me safe’ (boy, age 4) ‘Everyone has to look after each other’ (boy, age 4) ‘To feel safe, calm and happy and you always know friends are there for you’ (girl, age 7) ‘Being safe’ (boy, age 4)
**Caring environment (*n* = 4, 10.5%)**	**Feeling comfortable as home (*n* = 2, 5.3%)**
‘Everyone has to look after each other’ (boy, age 4) ‘That my teachers knows when I feel good or bad’ (boy, age 7) ‘Being kind and letting other people play your games. Not being too rough and just having other people with you’ (girl, age 7) ‘Kind people’ (girl, age unknown)	‘When you feel happy at school it makes you feel you are at home’ (girl, age 8) ‘That your seat belongs to you and all the stuff that you work on and use belongs to you’ (girl, age 7)

#### Visual responses

There were 22 drawings representing participants’ understanding of what constitutes belonging in their education and care setting. The five themes included: Happiness, Friendship/Togetherness, Love, Safety, and Inclusion. See Supplemental for samples of the visual responses. For happiness (*n* = 14, 63.6%), over half of the participant drawings included figures that were smiling and looked happy. These pictures also were often very colourful and included motifs like a shining sun or a blue sky. Two drawings included a rainbow. For friendship/togetherness (*n* = 13, 59.1%), multiple drawings exclusively depicted figures standing together and smiling, often holding hands. Most of the drawings depicted children with their friends, but two depicted a child with their parents or family, and one drawing depicted the child in class with their friends and teachers. For love (*n* = 5, 22.7%), some drawings either exclusively depicted love hearts or included these in the overall setting. Two of the drawings also included the word “love” in writing along with the heart symbols. Some of the drawings depicted the subject of the drawing inside a circle, representing safety (*n* = 4, 18.2%). In one of these safety drawings, the subject was inside a mountain looking out. For inclusion (*n* = 2, 9.1%), the drawings depicted groups of children together looking happy while another child stood alone looking sad. One of these drawings included a second scene where the child who had been alone had now joined the group and also looked happy.

#### Collective themes for understanding of belonging

**Figure 2.**
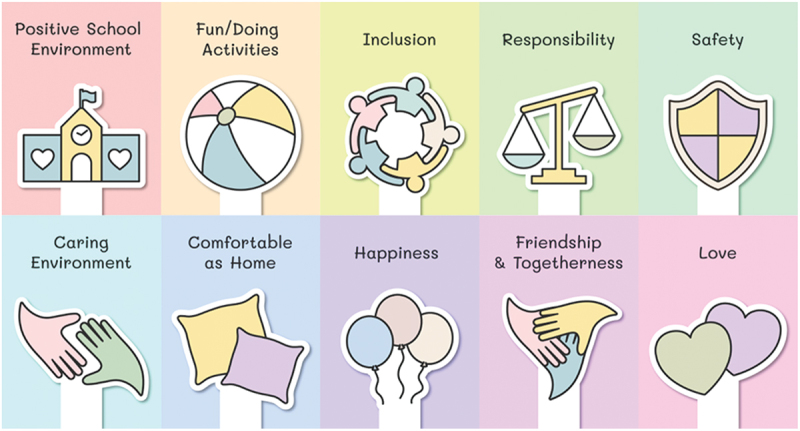
Children’s understanding of belonging in education and care contexts.

Taken together, there were 10 themes across the pictorial and textual data in the qualitative analysis of children’s understanding of belonging in ECEC and early years of school contexts. These collective themes are represented in Figure 2.

### Factors promoting belonging

To identify the factors that facilitate young children’s belonging in an ECEC and early years of context, we analysed children’s verbal responses, drawings, and explanations provided through the Draw, Write, Tell methodology. This involved examining the symbolic representations and social contexts depicted in the drawings, as well as the children’s verbal narratives, to identify recurring themes and insights into their understanding of belonging. There were 16 drawings uploaded, with 28 responses in total including verbal only responses. However, six participants used the same drawing to respond to both of the research questions.

The included themes relate to supportive relationships (e.g., positive teacher relationships, friendships), activities that promote engagement and inclusion (e.g., group play, enjoyable activities), and environmental factors (e.g., safety, caring spaces). These factors are considered as the elements or actions that create the conditions for belonging. Although there is overlap between the themes in this section and those in the previous section *Children’s Understanding of Belonging*, the goal of this section was to identify the conditions that facilitate or support children’s sense of belonging. These factors highlight the contextual and relational aspects that enable children to feel included, accepted, and safe, which in turn shapes their understanding of belonging. In the following descriptions, percentages of the sample were calculated separately for drawings and text responses given the unequal number of each type of response. See Supplemental for samples of the visual responses.

For Positive Experiences/Emotions (*n* = 20, 71.4%), most of the drawings (*n* = 14, 87.5%) depicted the participants as smiling and happy, and/or used symbols to communicate positive emotions such as a shining sun (*n* = 2, 12.5%) or love hearts (*n* = 2, 12.5%). Multiple drawings (*n* = 5, 31.3%) included extensive use of bright colours indicating positive emotions, with one participants’ explanation of their drawing stating “Belonging makes me feel like someone loves me … ” Verbal responses also indicated positive emotions such as “ … playing with my friends, it makes me feel happy” and “I am feeling the best”. In the theme of Social Inclusion and Friendship (*n* = 15, 53.6%) most of the drawings depicted subjects with a friend or friends (*n* = 14, 77.8%). In 12 (75%) of the drawings the figures are static and in two (12.5%) of the drawings they are playing together. A further two (12.5%) drawings included a heart motif indicating friendship. A similar number of verbal responses also included references to friendship or social inclusion, for example, “Me and my best friend Harry at school” and “If people were not mean and let me join in the games”. For Enjoyable Activities (*n* = 7, 25%) four drawings (25%) depicted participants engaging in activities they enjoy. Explanations for the drawings state “Going to the library, art work. Being in the library with my friends”, and “ … letting other people play our games … This is a picture of playing ball with another person”. Text only responses also mentioned “ … let(ting) me join in the games”, and “When I’m outside my friends help me build homes for tigers”.

Positive/Caring Teacher Relationships (*n* = 5, 17.9%) were depicted in three (18.8%) drawings, showing positive and caring relationships between participants and their teachers. In two (12.5%) of the drawings the subject is pictured with their teacher, and a further one (6.3%) depicts the student with the teacher and other students from the student’s class. Two further responses delivered verbally stated “My teacher and my friends”, and “My teachers help me”. For Helping Each Other (*n* = 4, 14.3%) the description for one (3.6%) of the drawings involves the concept of helping others (file failed to upload), stating “Everyone is in the jungle helping each other go up the hill … ” Additionally, the verbal response “When I’m outside my friends help me build homes for tigers” includes the concept of helping, as do the further responses “My teacher helps me” and “… and when I help the class pet called Lucie”. Safety (*n* = 3, 10.7%) was depicted in drawings showing subjects enclosed by a circle (*n* = 2, 12.5%) or a mountain (*n* = 1, 6.3%) indicating feelings of safety. One explanation for the drawings explicitly stated they were standing inside the mountain “because it was raining”. A further explanation also stated “Being in the library with my friends. The library is my safe space”. For Pets (*n* = 2, 7.1%), one (6.3%) drawing included a pet dog (given above under enjoyable activities), which the author explains saying “ … and there’s a dog because dogs kinda help you feel like you belong”. A further verbal response also stated “ … when I help the class pet called Lucie”.

#### Collective themes of factors promoting belonging

**Figure 3.**
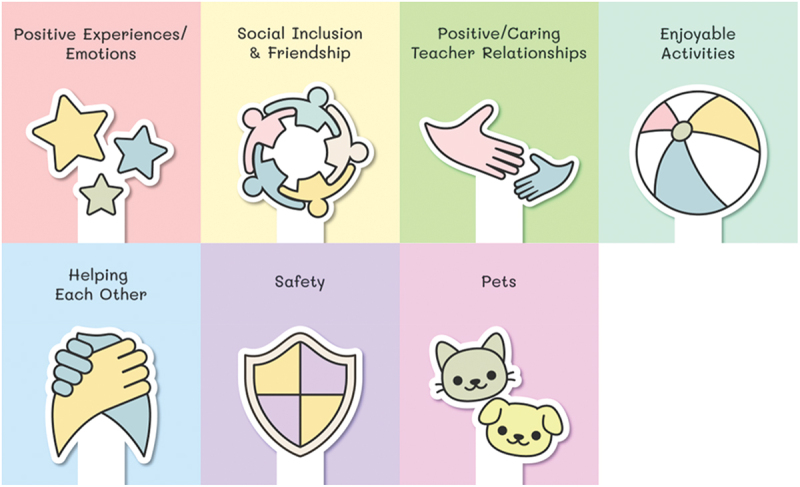
Facilitators of belonging in ECEC and early years of school contexts.

Taken together, there were seven themes across the pictorial and textual data in the qualitative analysis of factors children felt contributed to their sense of belonging in ECEC and early years of school contexts. This textual data refers to the written transcripts of the children’s verbal responses recorded by their parents and primary caregivers. These collective themes are represented in Figure 3.

## Discussion

The current study aimed to identify factors that promote belonging in children aged 3–8. This addresses a significant gap in existing research. The project, which identified a broad range of themes associated with what belonging means (i.e., feelings of happiness, friendship, togetherness, inclusion, safety, fun, responsibility to help belonging in others, and a loving, caring and comfortable environment) and what fosters belonging in young children (i.e., positive experiences and emotions, social inclusion, friendship, a sense of safety, positive and caring teacher relationships, and engaging in enjoyable activities), aligns with factors that have been previously identified in the broader school belonging literature, particularly in older age groups (Allen et al., 2023). This suggests that, like older children, belonging is a complex construct for young children, influenced by various emotional and social factors.

### How do young children experience belonging?

Participants’ answers in response to the question regarding how participants experience belonging in their education and care context indicate that for school (or other education and care contexts e.g., kindergarten, daycare), belonging consists of feelings of happiness, friendship, togetherness, inclusion, safety, fun, responsibility to help belonging in others, and a loving, caring, and comfortable environment. With the exception of feelings of happiness, safety, responsibility towards others, and a comfortable environment, these results are similar to those uncovered in previous research which found that belonging in early education and care settings involves being included in collective activities (Johansson, 2017), joint play, stable friendships and emotional bonding (Koivula & Hännikäinen, 2017), friendships, caring adults, and membership in the community (Einarsdottir et al., 2022). These findings confirm much of the previous research, which highlights the importance of inclusion, friendship, and emotional bonding in fostering belonging in ECEC contexts (Einarsdottir et al., 2022; Johansson, 2017; Koivula & Hännikäinen, 2017). However, the current study adds to this literature by showing that feelings of happiness, safety, and responsibility towards others, as well as the creation of a comfortable and nurturing environment, are also critical factors in children’s experiences of belonging.

### What helps facilitate belonging in young children?

In comparison to the factors involved in belonging in early education and care settings, participant responses regarding what helps them to feel like they belong in their ECEC and early years of school context indicate that positive experiences and emotions, social inclusion, friendship, a sense of safety, positive and caring teacher relationships, and engaging in enjoyable activities all help to promote belonging in their ECEC and early years of school context. Again, these results are similar to previous research which found that feelings of togetherness, safety and integrity, and a caring and loving approach (Eek-Karlsson & Emilson, 2023), a belief in diversity where children appreciate the different ways people do things, learn from each other, and feel valued (Gouldsboro, 2018), and cooperative communication style between educators and children (Ree & Emilson, 2020) all promote children’s sense of belonging in early education and care settings. However, the findings of positive experiences, emotions and fun, and engaging activities were unique to the current project.

Finally, an interesting finding was that participants often utilised similar drawings when responding to what helps them to feel a sense of belonging in their ECEC and early years of school context as they did for what belonging means in their ECEC and early years of school context. One participant explicitly stated that they did not produce another drawing to answer the second research question “because it would just be the same as the first one”. This, along with the previous research mentioned above (Eek-Karlsson & Emilson, 2023; Einarsdottir et al., 2022; Gouldsboro, 2018; Johansson, 2017; Koivula & Hännikäinen, 2017; Ree & Emilson, 2020) suggest that the factors involved in how participants perceive belonging in their early education and care context significantly overlap with what helps them feel that they belong in these settings.

### Practical recommendations for educators

Based on these findings, educators can foster belonging in early childhood education by creating an environment that is emotionally supportive, safe, and inclusive. A key strategy is designing learning experiences that are both collaborative and enjoyable, where the emphasis on play and fun helps cultivate positive emotions and engagement. Equally important is fostering a sense of safety, both physical and emotional. Establishing predictable routines, clear boundaries, and a nurturing environment helps children feel secure and confident in expressing their thoughts and emotions. Moreover, the responsibility children feel for helping others belong highlights the need to encourage prosocial behaviours and empathy. Educators can facilitate this by promoting cooperative activities where children support each other, reinforcing a collective sense of responsibility and inclusion. Lastly, building strong, positive relationships between teachers and children is essential. By being attuned to each child’s individual needs and maintaining open, caring communication with both children and their families, educators create a foundation of trust and connection that is crucial for fostering belonging in early educational settings.

### Limitations

The initial perspectives gathered in the study had certain limitations. While the choice of parents and primary caregivers as data collection agents allowed for a more comfortable and natural setting for young children, we acknowledge the potential for bias introduced by parent characteristics, including their own interpretations of belonging, their engagement in the study, and their expectations. This could influence how they guided their children or interpreted their responses. Future studies could benefit from exploring alternative methods of data collection, such as direct interviews with children or classroom-based observations, to address these limitations and ensure the reliability of the findings.

Additionally, the availability and capacity of parents and caregivers to participate potentially posed challenges, as their engagement required significant time and presence, reflected in the overall participation rate. Participant attrition, particularly among First Nations families, may have been influenced by time constraints and the commitment needed to complete the survey. Future studies should address these practical challenges to encourage sustained engagement and a more representative sample. This would also allow comparisons to be made between children and families of different cultural groups, something which was not possible for the current study given the diversity of the sample and small sample size. Future analyses may benefit from investigating whether any significant differences exist between these groups and the broader sample, particularly in terms of their perceptions of belonging. The need for broader representation from various Australian states and ECEC contexts also limits the generalisability of the findings. While the sample size was sufficient for qualitative analysis and theme saturation, it may not fully capture the diversity of experiences across the entire Australian population.

### Future research

Exploring the lived experiences of young children in their sense of belonging is a critical foundation for designing interventions and measures tailored to children aged 3 to 8 years (Faircloth et al., 2021). Such an approach, often underutilised underscores the importance of children’s firsthand perspectives. It shifts the focus to recognising children as right-holders of belonging, rather than simply stakeholders, aligning with the perspective of Canadian Indigenous scholar Jan Hare (2023). This approach also values children’s developing competencies and their unique insights into their environments. The findings of this study affirm young children’s capacity to provide meaningful contributions, offering a strong basis for future research constructing robust and reliable tools to measure and support belonging in early childhood contexts through professional development programs for educators and interventions for children. Future research should explore subgroup differences more deeply, particularly among neurodivergent children, children with disabilities, and First Nations children. Understanding how these groups uniquely conceptualise and experience belonging in early childhood education settings could lead to more inclusive practices that foster a stronger sense of belonging. Additionally, future studies could minimise the impact of priming by asking children to share their own ideas of belonging before any external prompts or explanations are provided. Last, accurately distinguishing between sex and gender is recognised as important in research (NHMRC, 2024). Future studies should refine measurement practices to better align with best-practice ensuring clarity in the conceptualisation and reporting of gender and sex in child demographic data of immense relevance to belonging research.

## Conclusion

When considered collectively, the findings of this project provide valuable insights into the conceptualisations of belonging by children aged 3–5. The results revealed that most participants in the sample comprehended the concept of belonging and associated it with positive emotional states, positive environment, friendship, safety, and inclusion, loving and caring environment, positive teacher interactions, fun and engaging activities, a kind and helping atmosphere, feeling comfort, and responsibility towards others. These findings hold immense significance in informing the development of future interventions and measures.

Honouring and valuing the voices of these young participants underscores their potential to offer profound insights into what shapes their own sense of belonging. This recognition highlights the importance of further research and continued efforts to listen to and learn from the perspectives of young children, as they play a vital role in shaping educational practices and policies that promote a strong sense of belonging in early childhood education.

## Supplementary Material

Supplemental Material

## Data Availability

Due to ethical restrictions and the young age of the participants, the data supporting this study cannot be made publicly available. Ethical approval for this research was granted on the condition that participant confidentiality would be strictly maintained, and data sharing would not be permitted. Requests for further information about the study may be directed to the corresponding author, subject to ethical considerations.

## Note

1. The wording of the item did not explicitly distinguish between sex and gender. Parents provided responses on behalf of their children, which may not reflect children’s self-identified gender identity.

## References

[cit0001] Allen, K. A., Berger, E., Reupert, A., Grove, C., May, F., Patlamazoglou, L., Gamble, N., Wurf, G., & Warton, W. (2023). Student-identified practices for improving belonging in Australian secondary schools: Moving beyond COVID-19. *School Mental Health*, 15(3), 927–13. 10.1007/s12310-023-09596-9

[cit0002] Angell, C., Alexander, J., & Hunt, J. A. (2015). ‘Draw, write and tell’: A literature review and methodological development on the ‘draw and write’ research method. *Journal of Early Childhood Research*, 13(1), 17–28. 10.1177/1476718X14538592

[cit0003] Australian Government Department of Education. (2022). *Belonging, being and becoming: The early years learning framework for Australia (V2.0)*. Australian Government Department of Education for the Ministerial Council. Retrieved September 9, 2023. https://www.acecqa.gov.au/sites/default/files/2023-01/EYLF-2022-V2.0.pdf

[cit0004] Baumeister, R. F., & Leary, M. R. (1995). The need to belong: Desire for interpersonal attachments as a fundamental human motivation. *Psychological Bulletin*, 117(3), 497–529. 10.1037/0033-2909.117.3.497 7777651

[cit0005] Braun, V., & Clarke, V. (2021a). Can I use TA? Should I use TA? Should I not use TA? Comparing reflexive thematic analysis and other pattern-based qualitative analytic approaches. *Counselling and Psychotherapy Research*, 21(1), 37–47. 10.1002/capr.12360

[cit0006] Braun, V., & Clarke, V. (2021b). One size fits all? What counts as quality practice in (reflexive) thematic analysis? *Qualitative Research in Psychology*, 18(3), 328–352. 10.1080/14780887.2020.1769238

[cit0007] Braun, V., & Clarke, V. (2024). Supporting best practice in reflexive thematic analysis reporting in palliative medicine: A review of published research and introduction to the reflexive thematic analysis reporting guidelines (RTARG). *Palliative Medicine*, 38(6), 608–616. 10.1177/02692163241234800 38469804 PMC11157981

[cit0008] Carter, M., McGee, R., Taylor, B., & Williams, S. (2007). Health outcomes in adolescence: Associations with family, friends and school engagement. *Journal of Adolescence*, 30(1), 51–62. 10.1016/j.adolescence.2005.04.002 16808970

[cit0009] Eek-Karlsson, L., & Emilson, A. (2023). Normalised diversity: Educators’ beliefs about children’s belonging in Swedish early childhood education. *Early Years*, 43(2), 317–331. 10.1080/09575146.2021.1951677

[cit0010] Einarsdottir, J., Juutinen, J., Emilson, A., Ólafsdóttir, S. M., Zachrisen, B., & Meuser, S. (2022). Children’s perspectives about belonging in educational settings in five European countries. *European Early Childhood Education Research Journal*, 30(3), 330–343. 10.1080/1350293X.2022.2055099

[cit0011] Emilson, A., & Eek-Karlsson, L. (2022). Doing belonging in early childhood settings in Sweden. *Early Child Development & Care*, 192(14), 2234–2245. 10.1080/03004430.2021.1998021

[cit0012] Faircloth, B. S., Gonzalez, L. M., Ramos, K., Hope, E. C., & Smith, C. D. (2021). *Resisting barriers to belonging: Conceptual critique and critical applications*. Oxford University Press.

[cit0013] Frydenberg, E., Care, E., Chan, E., & Freeman, E. (2009). Interrelationships between coping, school connectedness and well-being Erica Frydenberg. *Australian Journal of Education*, 53(3), 261–276. 10.1177/000494410905300305

[cit0014] Goodenow, C., & Grady, K. E. (1993). The relationship of school belonging and friends’ values to academic motivation among urban adolescent students. *The Journal of Experimental Education*, 62(1), 60–71. 10.1080/00220973.1993.9943831

[cit0015] Gouldsboro, J. M. (2018). *Promoting British values in the early years how to foster a sense of belonging*. Taylor and Francis.

[cit0016] Hare, J. (2023, August 17). Excellence and equity, connecting for equity and community, South Australian Secondary Principals Association (SASPA), SA Secondary Principals’ Association (SASPA) conference keynote presentation [video]. *YouTube*. https://www.youtube.com/watch?v=tveaTRi7cRU

[cit0017] Hudson, C., & Allen, K. A. (n.d.). *What gives children in their First Year of school a sense of school belonging?* Unpublished manuscript.

[cit0018] IBM Corp. (2020). *IBM SPSS statistics for windows, version 27.0*.

[cit0019] Johansson, E. (2017). Toddler’s Relationships: A Matter of Sharing Worlds. In L. Li, G. Quiñones, & A. Ridgway (Eds.), *Studying Babies and Toddlers. International Perspectives on Early Childhood Education and Development* (Vol. 20, pp. 13–27). Springer. 10.1007/978-981-10-3197-7_2

[cit0020] Johansson, E., & Rosell, Y. (2021). Social sustainability through children’s expressions of belonging in peer communities. *Sustainability*, 13(7), 1–17. 10.3390/su13073839

[cit0021] Koivula, M., & Hännikäinen, M. (2017). Building children’s sense of community in a day care centre through small groups in play. *Early Years*, 37(2), 126–142. 10.1080/09575146.2016.1180590

[cit0022] Koro-Ljungberg, M., & Cannella, G. S. (2017). Critical qualitative inquiry: Histories, methodologies, and possibilities. *International Review of Qualitative Research*, 10(4), 327–339. 10.1525/irqr.2017.10.4.327

[cit0023] Kuh, D. (2002). Mortality in adults aged 26-54 years related to socioeconomic conditions in childhood and adulthood: Post war birth cohort study. *BMJ*, 325(7372), 1076–1080. 10.1136/bmj.325.7372.1076 12424168 PMC131184

[cit0024] Langenberg, C., Kuh, D., Wadsworth, M. E., Brunner, E., & Hardy, R. (2006). Social circumstances and education: Life course origins of social inequalities in metabolic risk in a prospective national birth cohort. *American Journal of Public Health*, 96(12), 2216–2221. 10.2105/AJPH.2004.049429 17077402 PMC1698170

[cit0025] Lee, H., & Schafer, M. (2021). Are positive childhood experiences linked to better cognitive functioning in later life?: Examining the role of life course pathways. *Journal of Aging & Health*, 33(3–4), 217–226. 10.1177/0898264320972547 33228449 PMC7906946

[cit0026] Pope, N., Tallon, M., Leslie, G., & Wilson, S. (2019). Using ‘draw, write and tell’ to understand children’s health-related experiences. *Nurse Researcher*, 26(2), 42–45. 10.7748/nr.2018.e1594 30203931

[cit0027] Productivity Commission. (2020). *Mental health, report No. 95, Canberra*. https://www.pc.gov.au/inquiries/completed/mental-health/report

[cit0028] Ree, M., & Emilson, A. (2020). Participation in communities in ECEC expressed in child–educator interactions. *Early Child Development & Care*, 190(14), 2229–2240. 10.1080/03004430.2019.1566230

[cit0029] Ryzin, M. J., Gravely, A. A., & Roseth, C. J. (2009). Autonomy, belongingness, and engagement in school as contributors to adolescent psychological well-being. *Journal of Youth & Adolescence*, 38(1), 1–12. 10.1007/s10964-007-9257-4 19636787

[cit0030] Saunders, B., Sim, J., Kingstone, T., Baker, S., Waterfield, J., Bartlam, B., Burroughs, H., & Jinks, C. (2018). Saturation in qualitative research: Exploring its conceptualization and operationalization. *Quality & Quantity*, 52(4), 1893–1907. 10.1007/s11135-017-0574-8 29937585 PMC5993836

[cit0031] Scarf, D., Moradi, S., McGaw, K., Hewitt, J., Hayhurst, J. G., Boyes, M., Ruffman, T., & Hunter, J. A. (2016). Somewhere I belong: Long-term increases in adolescents’ resilience are predicted by perceived belonging to the in-group. *British Journal of Social Psychology*, 55(3), 588–599. 10.1111/bjso.12151 27448617

[cit0032] Sumsion, J., Harrison, L., Letsch, K., Bradley, B. S., & Stapleton, M. (2018). ‘Belonging’ in Australian early childhood education and care curriculum and quality assurance: Opportunities and risks. *Contemporary Issues in Early Childhood*, 19(4), 340–355. 10.1177/1463949118796239

[cit0033] Waters, L., Dussert, D., Martínez Sánchez, G., & Loton, D. (2022). Conceptualizations of well-being during middle childhood: Investigating developmental shifts through narrative analysis. In K. A. Allen, M. J. Furlong, S. M. Suldo, & D. A. Vella-Brodrick (Eds.), *The handbook of positive psychology in schools (third edition): In support of positive educational processes* (pp. 182–201). Routledge.

