# Supplementary material for: Belonging in their words: exploring early childhood perspectives using the draw, write, tell method
Source: Aust J Psychol. 2025 Feb 16;77(1):2463949. doi: 10.1080/00049530.2025.2463949 (PMC12218535; doi:10.1080/00049530.2025.2463949)
Supplement: Supplemental Material [file RAUP_A_2463949_SM9007.docx]

**Supplemental Information**

**Supplemental A: Social Script**

Sammy feels like they Belong

Hi, I'm Sammy! Sometimes I go to a place where I learn things. This place might be a school, a preschool, day care or a playgroup. This is a story about how I feel like I belong in my learning place.

Belonging means feeling safe, comfortable, and happy in a place or group. I feel accepted and cared for when I belong in my learning place. This means that people like me and listen to me. People here respect me and value me too. I feel at home when I am in my learning place!

There are adults in my learning place. They might be teachers, helpers, or even my own family. These adults are there to help me. I feel happy when I see them. I like the adults in my learning place. They are fair and kind.

There are other kids in my learning place. Some are my friends. The other kids play with me, talk to me, and make me feel included in groups. I feel accepted by others in my learning place. I can just be myself!

When I am in my learning place, I feel safe. But sometimes I feel like I don't belong. When I feel lonely or left out, the adults in my learning place help. They help me feel like I fit in and belong again.

I know that I belong in my learning place. Tomorrow, I will go back and belong all over again.

**Supplemental B: Results of Priming Questions**

The four priming questions were designed to help the young participants become familiar with discussing the concept of belonging. The priming questions were designed to elicit general reflections about belonging to help prepare and scaffold children’s understanding of belonging so that it could be applied to their ECEC and early years of school contexts. Specifically, the priming questions were delivered to the participants immediately before delivering the survey questions regarding what they understand belonging to be in their ECEC or early years of school context and what helps them feel like they belong in this setting in order to maximise their responses.

When asked the first priming question ‘Have you felt like you belonged before?’, the majority of children and students (*n* = 25, 61.9%) indicated that they had felt a sense of belonging, with only one (2.6%) indicating they had not. Several children (*n* = 12, 36%) gave more equivalent responses to the first question. For example, some were less certain or ambiguous about their sense of belonging (e.g., 'I don't know' or 'sometimes not but sometimes I do'), or indicated that their neurodivergent status impacted on their sense of belonging (e.g., ‘my brain doesn’t work like that’).

Responses to the further priming question ‘What are some of the places you feel like you belong?’ generally involved more than one setting. Whilst 28 (73.7%) of the children indicated that they felt a sense of belonging in their home, 21 participants (55.3%) responded that they felt they belonged both at home and in their ECEC or early years of school setting, and 16 participants (42.1%) indicated that they felt a sense of belonging at their home, their ECEC or early years of school setting and another setting such as their previous ECEC or school setting, being with their parents and primary caregivers or family, at their grandparents or other relatives’ house, being with their friends or at their friend’s house, in the family car, engaging in after school activities, at church, in a specific room, when playing sport, when playing with their toys, when they are on holidays, or a combination of these. Singular responses to the primer question included ‘At kindy’ (*n* = 1, 2.6%), ‘Classroom’ (*n* = 1, 2.6%), ‘Home’ (*n* = 1, 2.6%), and ‘India’ (*n* = 1, 2.6%).

When responding to the question ‘How does belonging make you feel?’, nearly all of the participants (*n* = 36, 94.7%) associated positive emotions with belonging. The most common terms used by participants to describe belonging were ‘happy’ (*n* = 17, 44.7%), ‘good’ (*n* = 7, 18.4%), ‘safe’ (*n* = 3, 7.9%), ‘nice’ (*n* = 3, 7.9%), and ‘excited’ (*n* = 3, 7.9%). Some children (*n* = 4) described belonging as a positive experience such as feeling comfortable, normal, calm, and fuzzy. While others (*n* = 7) offered more complex descriptions of belonging including like being cared for, experiencing a sense of meaning, feeling like sitting in a pile of feathers, being included and not left out, a love heart, and like having something all to myself, or wanting to play.

Finally, responses to the primer question, ‘Can you explain what you think belonging means, in your own words?’ indicated that nearly all of the participants (*n* = 37, 97.4%) understood the concept of belonging. The most common response (*n* = 11, 28.9%) was again to associate belonging with happiness. Further responses associated belonging with friendship or being with friends (*n* = 7, 18.4%), love (*n* = 6, 15.8%), safety (*n* = 5, 13.2%), a place where participants liked being (*n* = 5, 13.2%), being included (*n* = 3, 7.9%), and where participants feel at home (*n* = 3, 7.9%), calmness (*n* = 3, 7.9%), togetherness (*n* = 3, 7.9%), being looked after or cared for (*n* = 2, 5.3%), not being alone (*n* = 2, 5.3%), being listened to (*n* = 2, 5.3%), being with their parents and primary caregivers (*n* = 1, 2.6%), participating in activities that they enjoy (*n* = 1, 2.6%), their toys or possessions (*n* = 1, 2.6%), equality (*n* = 1, 2.6%), and kindness (*n* = 1, 2.6%). Only one (2.6%) participant indicated that they either did not understand the concept of belonging or lacked the ability to articulate it: ‘I don’t know how to explain it’ (*n* = 1, 2.6%).

**Supplemental C: Visual Responses of Understanding Belonging**

| **Happiness**  **Figure A Figure B** | |
| --- | --- |
| 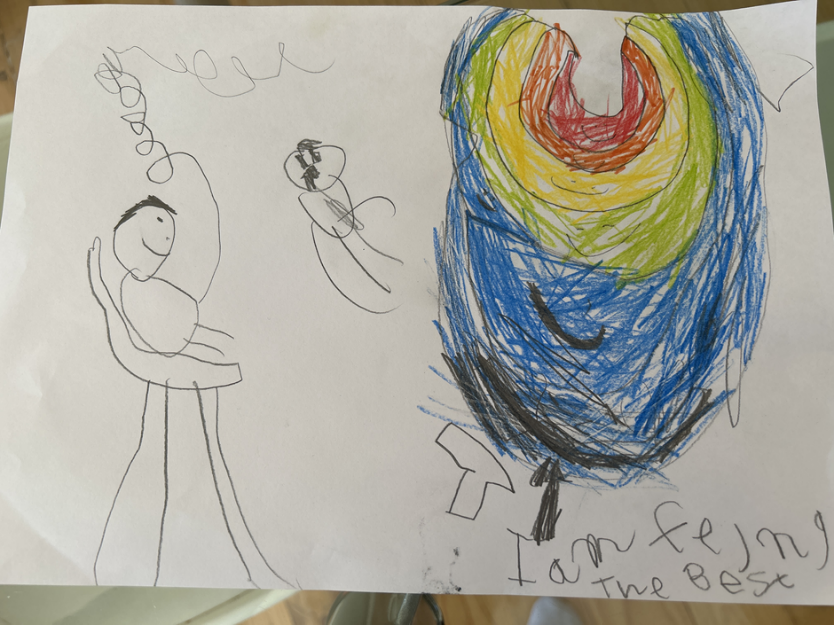 | 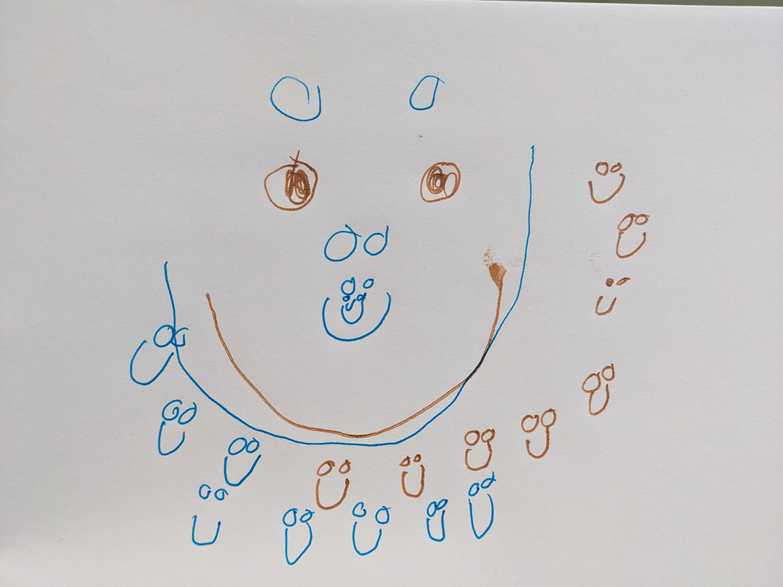 |
| **Friendship/Togetherness**  **Figure C Figure D** | |
| 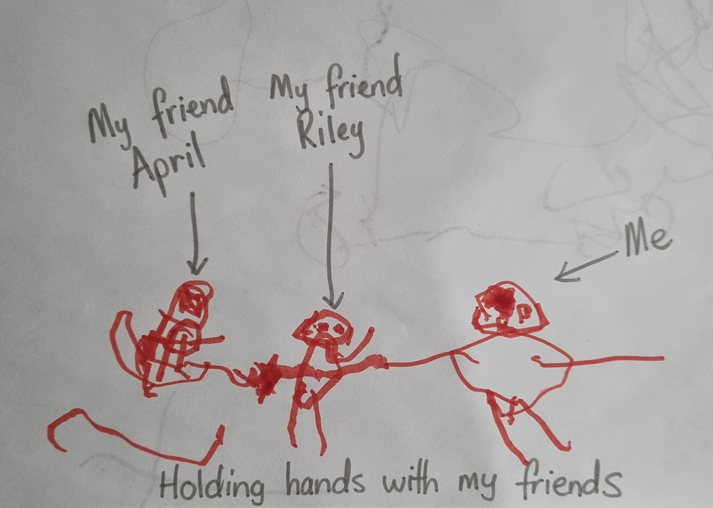 | 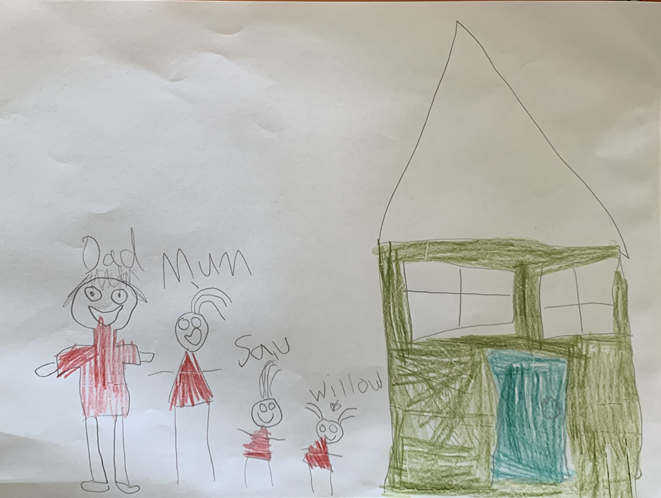 |
| **Love**  **Figure E Figure F** | |
| 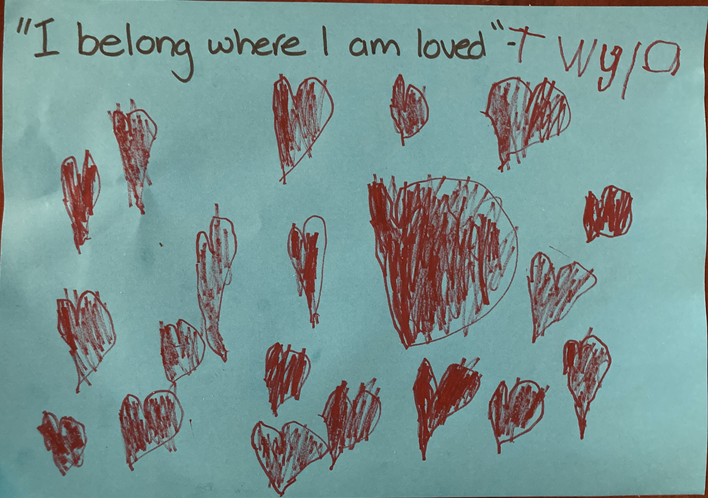 | 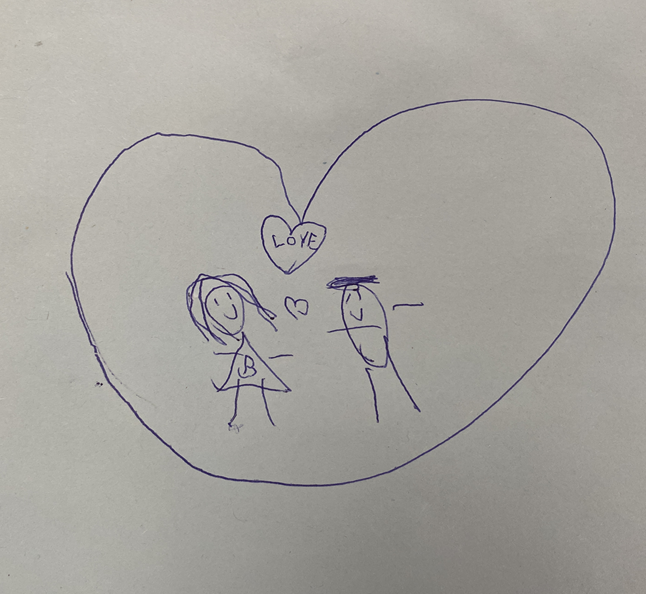 |
| **Safety**  **Figure G Figure H** | |
| 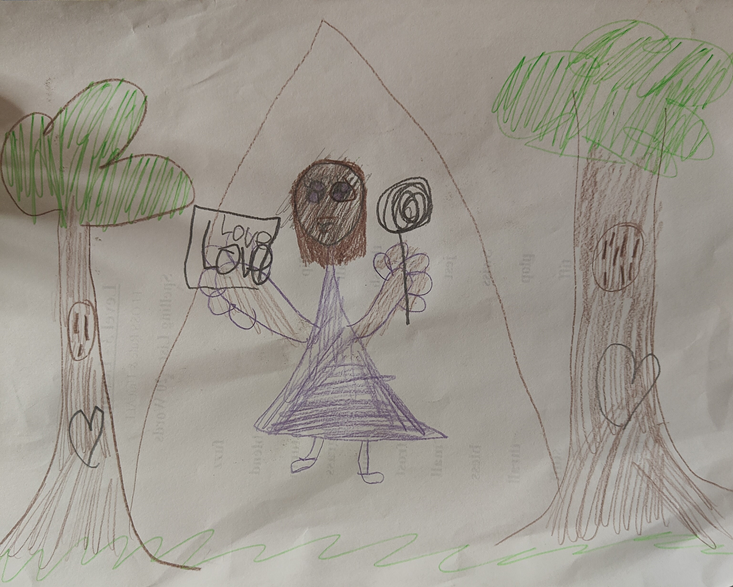 | 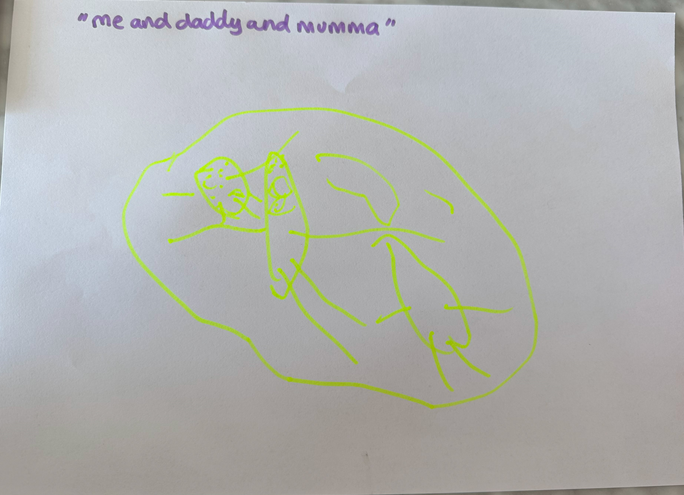 |
| **Inclusion**  **Figure I Figure J** | |
| 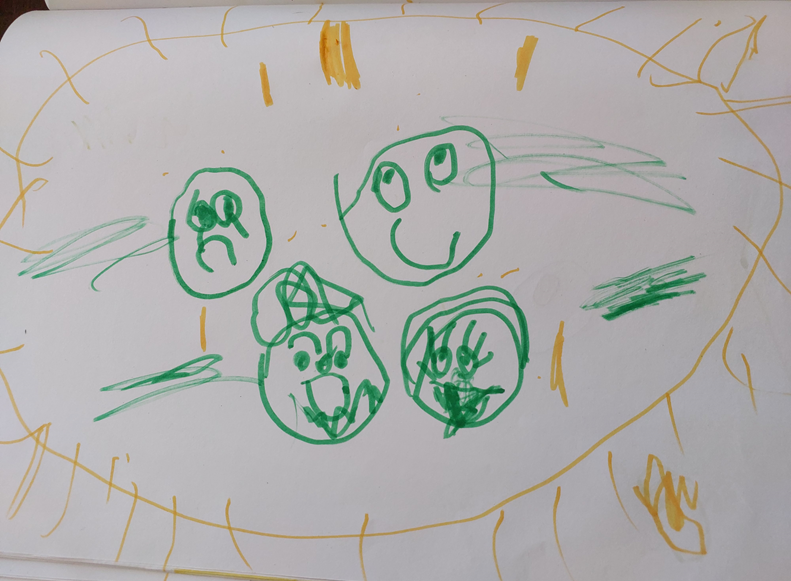 | 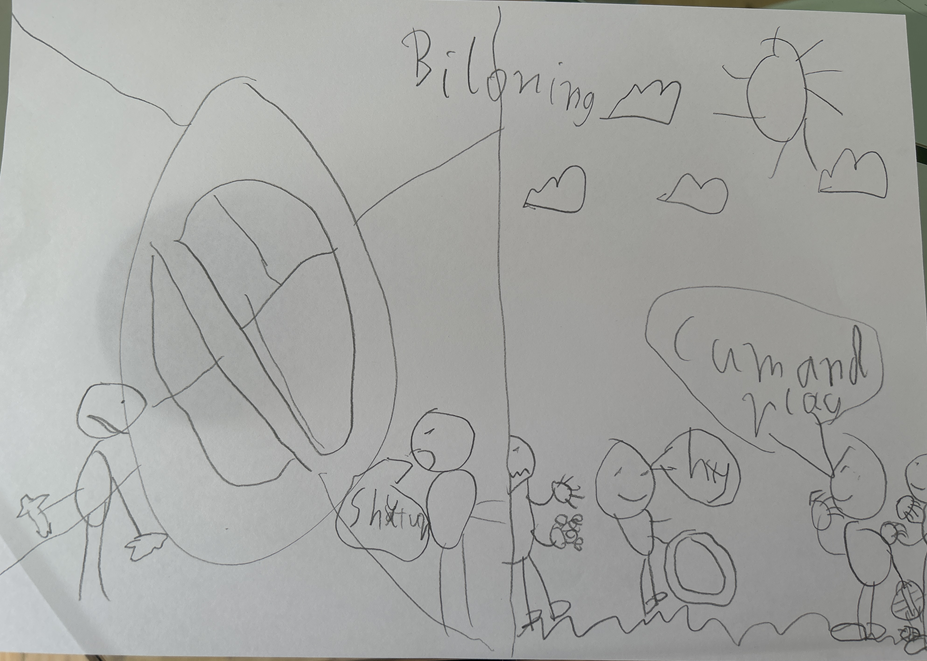 |

**Supplemental D:** **Visual Responses of Factors Promoting Belonging**

| **Positive Experiences/Emotions**  **Figure K Figure L** | |
| --- | --- |
| 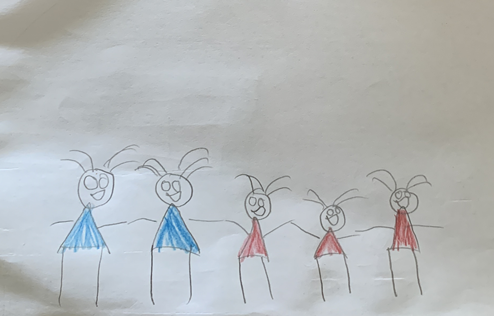 | 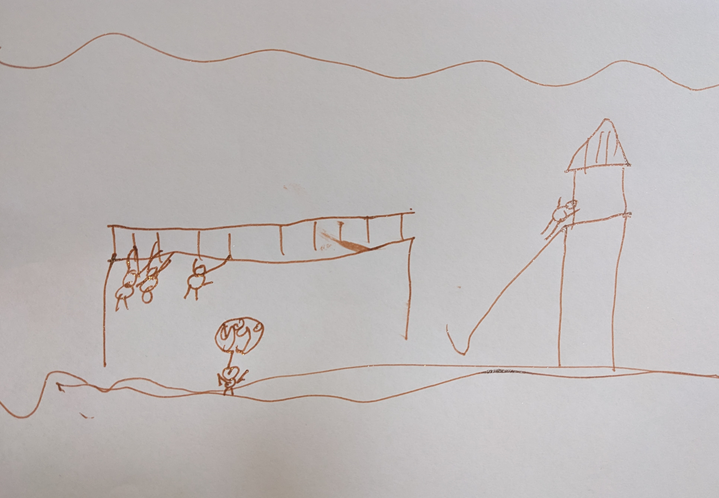 |
| **Social inclusion and Friendship**  **Figure M Figure N** | |
| 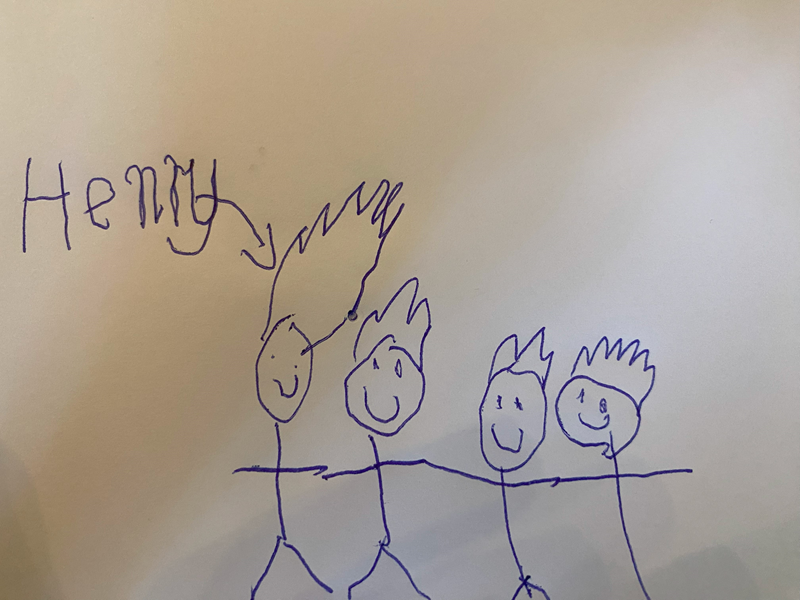 | 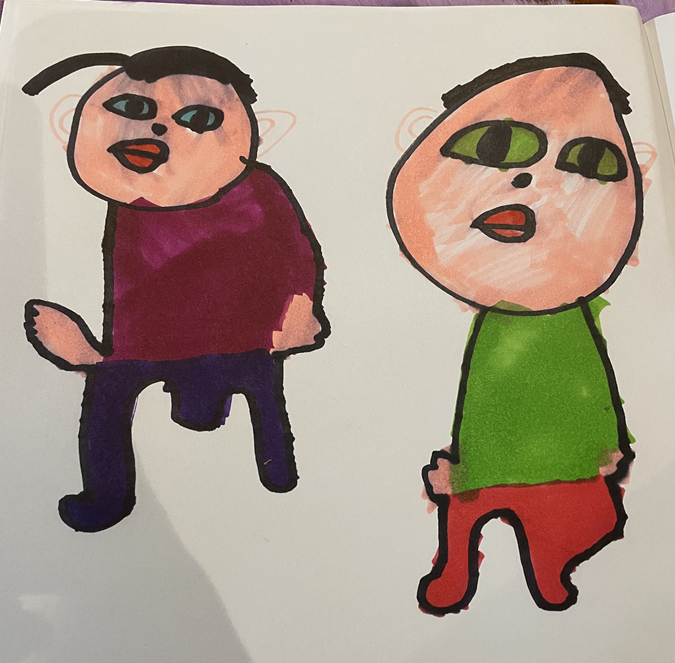 |
| **Enjoyable Activities**  **Figure O Figure P** | |
| 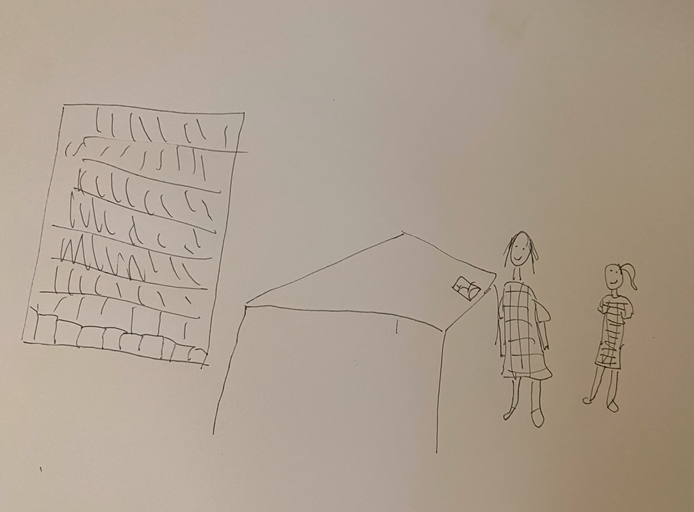 | 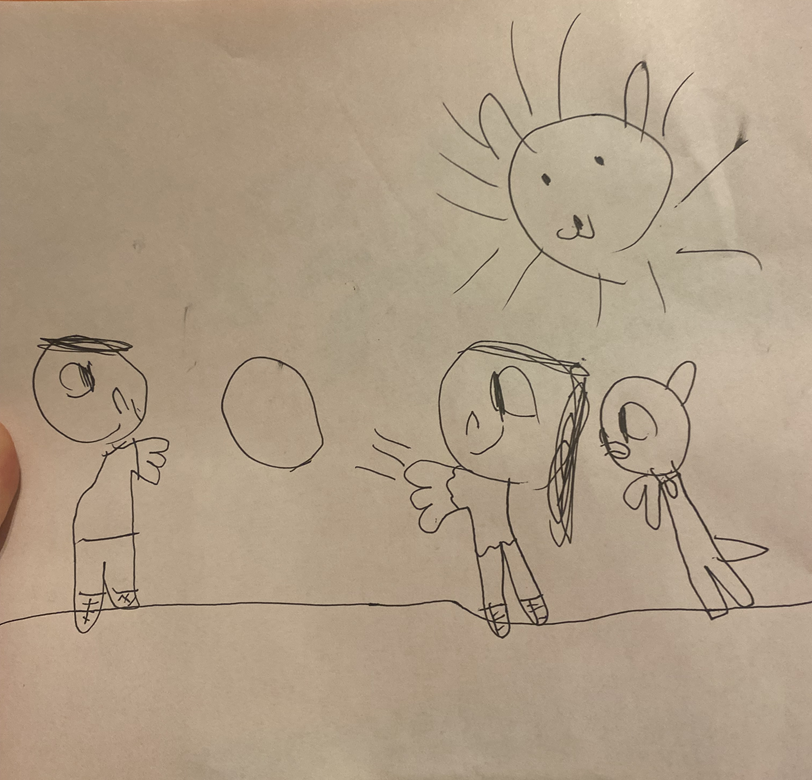 |
| **Positive/Caring Teacher Relationships**  **Figure Q Figure R** | |
| 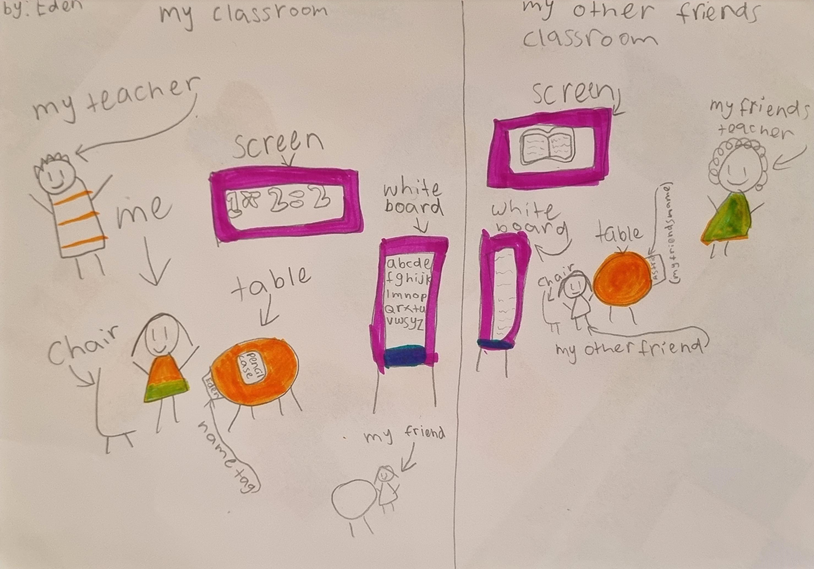 | 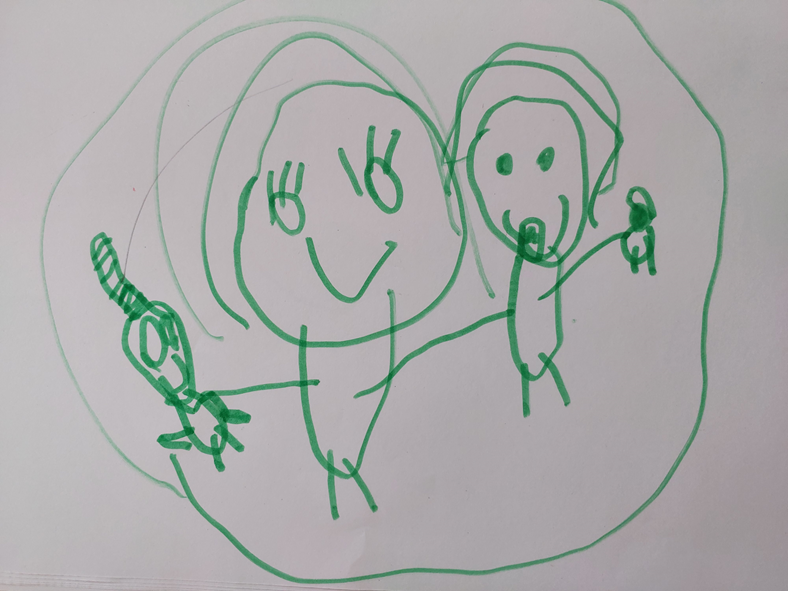 |
| **Safety**  **Figure S** | |
| 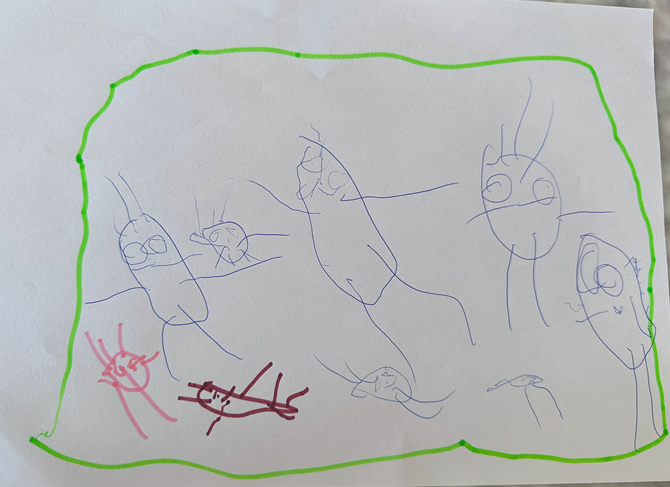 |  |
